# Islet Stellate Cells Regulate Insulin Secretion via Wnt5a in Min6 Cells

**DOI:** 10.1155/2020/4708132

**Published:** 2020-02-24

**Authors:** Wei Xu, Peter M. Jones, Houfa Geng, Rui Li, Xuekui Liu, Yinxia Li, Qian Lv, Ying Liu, Jie Wang, Xiuli Wang, Zilin Sun, Jun Liang

**Affiliations:** ^1^Department of Endocrinology, Xuzhou Central Hospital, Xuzhou Institute of Medical Sciences, Xuzhou Clinical School of Nanjing Medical University, Affiliated Hospital of Medical School of Southeast University, Xuzhou, Jiangsu, China; ^2^Diabetes Research Group, Division of Diabetes & Nutritional Sciences, School of Medicine, King's College London, London, UK; ^3^Department of Endocrinology, Zhongda Hospital, Institute of Diabetes, Medical School, Southeast University, Nanjing, China

## Abstract

**Background:**

Type 2 diabetes mellitus is a serious public health problem worldwide. Accumulating evidence has shown that *β*-cell dysfunction is an important mechanism underlying diabetes mellitus. The changes in the physiological state of islet stellate cells (ISCs) and the effects of these cells on *β*-cell dysfunction is an important mechanism underlying diabetes mellitus. The changes in the physiological state of islet stellate cells (ISCs) and the effects of these cells on

**Methods:**

Glucose-stimulated insulin secretion (GSIS) from Min6 cells was examined by estimating the insulin levels in response to high glucose challenge after culture with ISC supernatant or exogenous Wnt5a. Western blotting and quantitative real-time polymerase chain reaction (qRT-PCR) analyses were used to observe changes in the *β*-cell dysfunction is an important mechanism underlying diabetes mellitus. The changes in the physiological state of islet stellate cells (ISCs) and the effects of these cells on

**Results:**

We observed a significant increase in insulin secretion from Min6 cells cocultured in vitro with supernatant from db/m mouse ISCs compared to that from Min6 cells cocultured with supernatant from db/db mouse ISCs; The intracellular Ca^2+^ concentration in Min6 cells increased in cultured in vitro with supernatant from db/m mouse ISCs and exogenous Wnt5a compared to that from control Min6 cells. Culture of Min6 cells with exogenous Wnt5a caused a significant increase in pCamKII, pFoxO1, PDX-1, and Glut2 levels compared to those in Min6 cells cultured alone; this treatment further decreased Ror2 and Cask expression but did not affect *β*-cell dysfunction is an important mechanism underlying diabetes mellitus. The changes in the physiological state of islet stellate cells (ISCs) and the effects of these cells on

**Conclusion:**

ISCs regulate insulin secretion from Min6 cells through the Wnt5a protein-induced Wnt-calcium and FoxO1-PDX1-GLUT2-insulin signalling cascades.

## 1. Introduction

Type 2 diabetes mellitus (T2DM) is a common metabolic disorder that is currently a serious public health problem. Insulin resistance and/or insufficient insulin secretion are pathogenic mechanisms of diabetes. Dysfunction of *β*-cells leads to a decrease in insulin secretion, which plays an important role in the development of diabetes [1]. The effect of newly discovered cells such as stellate cells on *β*-cell insulin secretion in the islet microenvironment has been a popular focus of islet function research. Undoubtedly, understanding the basic mechanisms of *β*-cell insulin secretion by islet stellate cells (ISCs) is essential to further elucidate the regulation of *β*-cell function and the prevention and treatment of diabetes.

Various cells in islets affect the function of *β*-cells through the paracrine effects of hormones, such as somatostatin (*δ* cells) [2, 3], glucagon (*α* cells) [4], and pancreatic polypeptide (PP cells) [5], which also regulate insulin synthesis and secretion. Recent studies have found that in addition to *δ*-cells, *α*-cells, and PP cells, other interstitial cells such as fibroblasts and vascular endothelial cells are present in islets and may affect *β*-cell function. Vascular endothelial cells participate in pancreatic development and affect islet morphology and function [6]. Previous studies showed that insulin secretion from islets cocultured in vitro with quiescent ISCs from db/m mice is significantly increased compared to that from islets cultured with activated ISCs from db/db mice and revealed that ISCs regulate *β*-cell function via Wnt5a [7]. However, the mechanism by which ISCs mediate *β*-cell insulin secretion homeostasis via Wnt5a has not been fully elucidated. Understanding the mechanism by which stellate cells regulate islet function via Wnt5a can help us classify crosstalk between cells inside the islets and the pathogenesis of diabetes.

Wnt5a, a member of the Wnt family, is involved in proper islet formation and insulin-mediated cell migration during pancreatic development in vertebrates [8–11]. The noncanonical pathway mediated by the Wnt5a protein can affect the occurrence of diabetes by inhibiting fat formation and obesity through the inhibition of PPAR*γ* and C/EBP in preadipocytes [12,13]. Previous studies confirmed that vascular insulin resistance in the arterioles of visceral adipose tissue in obese subjects is associated with upregulated Wnt5a-JNK signalling [14]. Secreted frizzled-related protein 5 (Sfrp5) has been shown to antagonize the Wnt/*β*-catenin signalling pathway, reducing *β*-cell insulin secretion by inhibiting the noncanonical Wnt signalling pathway [15]. In vivo experiments showed that Wnt5a stimulated insulin secretion in wild-type but not LRP-5^−/−^ mice, suggesting that Wnt5a contributes to glucose-induced insulin secretion in islets [16]. A clinical study by our team showed that the serum Wnt5a level was significantly decreased in newly diagnosed T2DM patients compared with normal controls [17]. In addition, exogenous Wnt5a inhibited the activation of ISCs and increased insulin secretion from islets [7,18]. The FoxO1 and Pdx1-Glut2-insulin pathway plays an important role in glucose-stimulated insulin secretion (GSIS) [19]. Wnt5a increased the expression of the maturation marker Glut2 after coculture with mouse islets and Min6 cells [20]. Previous studies of Wnt5a and diabetes focused on islet development, insulin resistance, inflammation, and studies relating the Wnt5a antagonist protein Sfrp5 to islet function. However, the mechanisms underlying the associations between Wnt5a and *β*-cell insulin secretion are unclear. Understanding the mechanism by which Wnt5a regulates *β*-cell insulin secretion can help clarify the effect of the noncanonical Wnt pathway on islet function maintenance.

The individual pathophysiological roles of ISCs have been extensively investigated, but the roles of ISCs and Wnt5a in the regulation of insulin secretion are unclear. Our study aimed at defining the specific mechanism by which ISCs promote insulin secretion in Min6 cells via the Wnt5a protein.

## 2. Materials and Methods

### 2.1. Animals

Male db/db mice (8, 20, and 28 weeks old) and sex-matched male db/m mice were purchased from the Model Animal Research Centre of Nanjing University (Nanjing, China). All animal procedures were approved by our Institution's Ethics Committee and carried out under a licence.

### 2.2. Hematoxylin and Eosin Staining

After the pancreas tissue sections were dewaxed, hydrated, and stained in hematoxylin solution for 5 min, they were decolorized in 1% hydrochloric acid ethanol for 10 sec. Sections were then stained with eosin for 2 min. After sealing the slides with a neutral resin, they were observed under light microscopy.

### 2.3. Immunohistochemistry

To confirm the Wnt5a protein expression in pancreatic samples (from 8, 20, and 28-week-old mice), immunohistochemistry was performed as described previously [7]. Mouse pancreases were fixed in 4% paraformaldehyde in 0.1M PBS for 24 h at 4°C, embedded in paraffin, and sectioned. Formalin-fixed, paraffin-embedded tissue sections were blocked with 5% bovine serum albumin (BSA) for 30 min and incubated overnight at 4°C with a rabbit anti-mouse Wnt5a and insulin antibody (1 : 200, Abcam, Cambridge, UK). The sections were then washed and incubated with HRP-conjugated anti-rabbit serum at room temperature for 30 min. The sections were then developed with DAB, counterstained with hematoxylin, and examined via microscopy.

### 2.4. Immunofluorescence Microscopy of Wnt5a, Frizzled5, Insulin, Glucagon, and Desmin

Immunofluorescence microscopy was performed as described previously [7] to evaluate the expression of Wnt5a and its receptor Frizzled5 (Fzd5) in Min6 cells, and insulin, glucagon, and desmin in islets. Immunostaining was performed with polyclonal antibodies specific for Wnt5a, Fzd5, insulin, glucagon, and desmin (1 : 200, Abcam, Cambridge, UK). All immunocytochemical analyses were performed in triplicate.

### 2.5. Isolation and Culture of Mouse ISCs

Mouse islets were isolated from mice (8 weeks old) by type IV collagenase (1 mg/ml; Sigma, CA, USA) digestion of the exocrine pancreas followed by purification on Histopaque 1077 (Sigma, CA, USA) density gradients. The isolated islets were cultured in RPMI-1640 medium containing 10% (v/v) foetal bovine serum (FBS) and penicillin-streptomycin for up to 48 h at 37°C, and the majority of islets attached to the dish within 7 days. “Passage 0” was defined as the time when the cultures were nearly confluent with ISCs. Cells were maintained in Dulbecco's modified Eagle's medium (DMEM)/Ham's F12 (1 : 1 v/v) (Sigma, CA, USA) containing 10% (v/v) FBS and used at passages 3–8. Min6 cells were divided into groups and cultured alone, with ISC supernatant or with exogenous Wnt5a (0.05 *μ*g/ml) (Wnt5a; R&D Systems, UK) for 48 h unless otherwise specified.

### 2.6. Apoptosis Assay

A Caspase-Glo 3/7 (Caspase-Glo®, Promega) apoptosis detection kit was used to analyse Min6 cell apoptosis as described previously [7]. Groups of 2 × 10^4^ Min6 cells were maintained in culture in DMEM containing 2% FBS in the absence or presence of exogenous Wnt5a (0.05 *μ*g/ml) for 48 h and were subsequently incubated for 20 h in the absence or presence of a cytokine cocktail (1 U/*μ*l TNF-*α*, 0.05 U/*μ*l IL-1*β*, and 1 U/*μ*l IFN-*γ*) to induce apoptosis. Apoptosis was assessed by measuring caspase-3/7 activity as described previously [7].

### 2.7. Determination of Cell Proliferation

To confirm Min6 cell proliferation, a 5-ethynyl-2′-deoxyuridine (EdU) assay was used for analysis as described previously [18]. Groups of 2 × 10^4^ Min6 cells were incubated in the absence or presence of exogenous Wnt5a (0.05 *μ*g/ml) for 48 h before labelling with 10 *μ*M EdU for 4 h at 37°C, and cell proliferation was assessed by colorimetric quantification of EdU incorporation into cellular DNA [18].

### 2.8. In Vitro Secretory Function Assay in Min6 Cells

Each group of Min6 cells was divided into cells cultured alone, cells cultured with ISC supernatant, and cells cultured with exogenous Wnt5a (0.05 *μ*g/ml) for 48 h. GSIS from Min6 cells was examined as described previously, insulin was measured in GEY & GEY buffer with 2 mM glucose, and 20 mM glucose was added to each well [7]. Insulin content was measured by radioimmunoassay [7].

### 2.9. Measurement of the Intracellular Ca^2+^ Concentration

Flow cytometry was used to confirm the intracellular Ca^2+^ concentration in Min6 cells. Min6 cells were divided into groups and cultured alone, cultured with ISC cell supernatant, or cultured with exogenous Wnt5a (0.05 *μ*g/ml) for 48 h. Then, the cells were trypsinized, washed, and incubated in complete DMEM containing 5 *μ*M Fluo-4-acetoxymethyl ester (Fluo-4 AM; Molecular Probes, BD, USA) and 3% dimethyl sulfoxide at 37°C for 20 min. After incubation, the cells were washed three times with PBS by centrifugation. The cells were then resuspended in PBS. For each sample, fluorescence was analysed using a 530/30 filter.

### 2.10. Quantitative Real-Time Polymerase Chain Reaction (qRT-PCR)

Min6 cells were seeded into dishes and cultured alone, with ISC culture supernatant, or with exogenous Wnt5a (0.05 *μ*g/ml) for 48 h, unless otherwise specified. After 48 h, qRT-PCR was performed as described previously [7]. The qRT-PCR experiments were repeated at least 3 times.

The PCR primer sequences were as follows (Table 1).

### 2.11. Western Blotting Analysis

Min6 cells were divided into groups and cultured alone, with ISC cell supernatant, or with exogenous Wnt5a (0.05 *μ*g/ml) for 48 h. After 48 h, western blotting was performed [11] with the primary antibodies specific for the following proteins: Wnt5a (1 : 3000, Abcam, UK), *β*-catenin (1 : 1000, Proteintech, China), receptor tyrosine kinase-like orphan receptor 2 (Ror2) (1 : 1000, Santa Cruz, USA), phosphorylated Ca(2+)/calmodulin (CaM)-dependent protein kinase II (CamKII) (1 : 2000, Abcam, UK), FoxO1 (1 : 3000, Abcam, UK), pFoxO1 (1 : 3000, Abcam, UK), PDX1 (1 : 3000, Abcam, UK), Glut2 (1 : 3000, Abcam, UK), CASK (1 : 2000, Abcam, UK), and GAPDH (1 : 5000, Sigma, USA).

### 2.12. Statistical Analysis

The results are shown as the mean ± SEM for quantitative data. Multiple comparison analyses were performed in SAS using a Bonferroni's *t*-test, and differences were considered significant when *p* < 0.05.

## 3. Results

### 3.1. Insulin Secretion in Islets Is Increased by Coculture with ISCs

**Figure 1:**
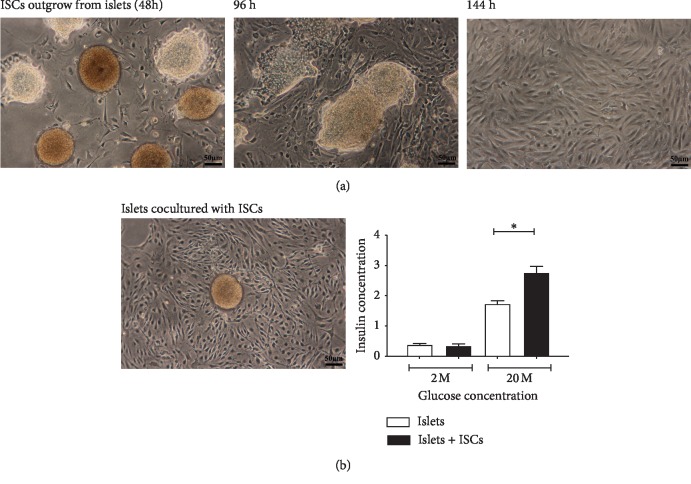
Insulin secretion in islets is increased by coculture with ISCs. (a) Islets were isolated from db/m mice, and ISCs outgrowth from the islets was analysed by microscopy (scale bars = 50 *μ*m). (b) Compared with islets cultured alone, islets cocultured with ISCs isolated from db/m mice showed significantly increased insulin secretion in vitro. The data are expressed as the mean ± SE (*n* = 3), ^*∗*^*p* < 0.05, ^*∗∗*^*p* < 0.01, vs. islets cultured alone.

ISC outgrowth from islets increased with increasing culture duration (Figure 1(a)). Insulin secretion was significantly increased in islets cocultured in vitro with ISCs compared to those cultured alone (Figure 1(b)).

### 3.2. Expression of Wnt5a in the Pancreas and Min6 Cells

**Figure 2:**
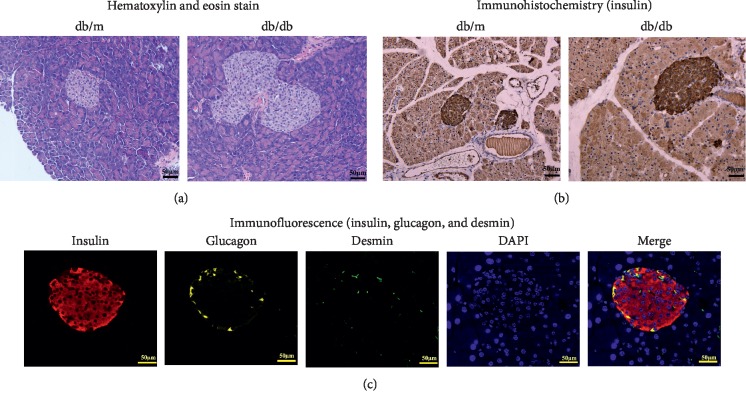
Expression of insulin, glucagon, and desmin in the islets. (a) Hematoxylin and eosin staining of pancreas tissue sections (scale bar = 50 *μ*m). (b) Paraffin-embedded sections of db/m and db/db mouse pancreases showing the expression of insulin as determined by immunohistochemistry (scale bar = 50 *μ*m). (c) Immunofluorescence staining of islet for insulin, glucagon, desmin, and DAPI (scale bar = 50 *μ*m).

**Figure 3:**
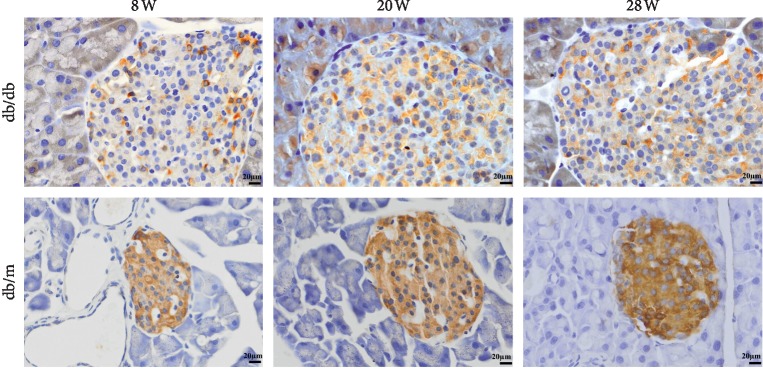
Expression of Wnt5a in the pancreas. Paraffin-embedded sections of db/m and db/db mouse pancreases showing the expression of Wnt5a as determined by immunohistochemistry (scale bar = 50 *μ*m).

**Figure 4:**
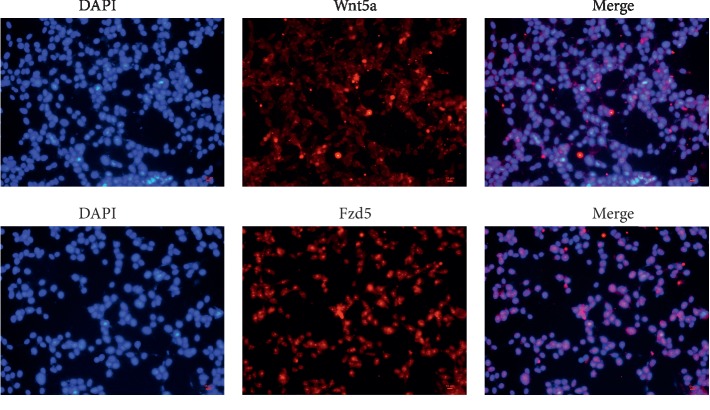
Expression of Wnt5a and Fzd5 in the Min6 cells. Immunofluorescence staining of Min6 cells for Wnt5a and Fzd5 (scale bar = 10 *μ*m).

The islet area of pancreatic tissues from db/m mice was significantly smaller than that of db/db diabetic mice. The shape of the islets was regular and appeared round or elliptical. The islet morphology of db/db mice was irregular (Figure 2(a)). The expression of insulin, glucagon, and desmin was detected in islets by immunofluorescence microscopy measurements (Figures 2(b) and 2(c)). Wnt5a protein expression was detectable by immunohistochemistry in pancreatic sections, as shown in Figure 3. Heavily immunostained Wnt5a^+^ islets were observed in the pancreases of db/m mice. In contrast, pancreases from db/db mice contained lightly stained Wnt5a^+^ islets. The expression of Wnt5a and Fzd5 was detected in Min6 cells by immunofluorescence microscopy measurements (Figure 4). Together, these data demonstrated that the Wnt5a protein plays an important role in islet morphology and functional maintenance.

### 3.3. Wnt5a Increases Insulin Secretion from Min6 Cells

**Figure 5:**
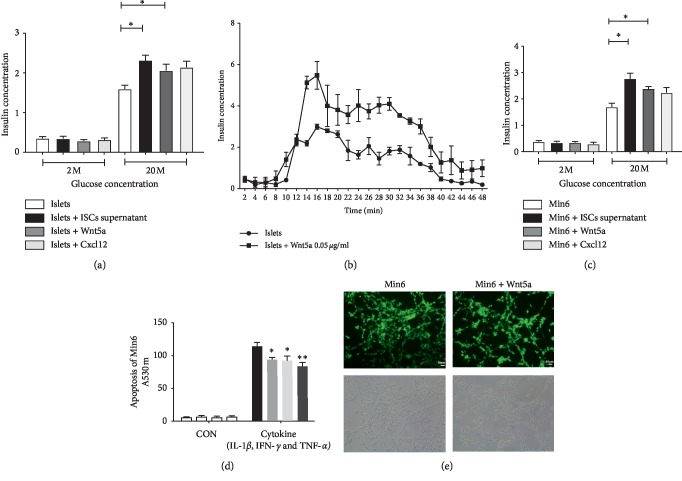
Wnt5a increases insulin secretion from Min6 cells. (a, b) Compared with islets cultured alone, islets cocultured with ISCs or Wnt5a exhibited significantly increased insulin secretion. The data are expressed as the mean ± SE (*n* = 3); ^*∗*^*p* < 0.05, ^*∗∗*^*p* < 0.01, vs. islets cultured alone. (c) Compared with Min6 cells cultured alone, Min6 cells cocultured with ISCs or Wnt5a exhibited significantly increased insulin secretion. (d) Apoptosis assays of Min6 cells from the cytokine-induced and control groups. (e) Proliferation assays of Min6 cells from the cytokine-induced and control groups. The data are expressed as the means ± SE (*n* = 3); ^*∗*^*p* < 0.05, ^*∗∗*^*p* < 0.01, vs. Min6 cells cultured alone.

The effects of exogenous Wnt5a on insulin secretion were investigated by incubating islets (static incubation and perifusion) and Min6 cells (static incubation) for 1 h with substimulatory (2 mM) or maximal stimulatory (20 mM) concentrations of glucose in the absence or presence of 0.05 *μ*g/ml exogenous Wnt5a as shown in Figure 5. Treatment with exogenous Wnt5a under 2 mM glucose conditions did not affect basal insulin secretion from islets and Min6 cells that had been pretreated for 48 h but significantly potentiated 20 mM glucose-induced insulin secretion (Figures 5(a) and 5(c)). This effect was reproducible, and sufficiently similar results were demonstrated in islets under perifusion conditions (Figure 5(b)).  Figure 5(d) shows that preculture for 48 h in the presence of 0.05 *μ*g/ml exogenous Wnt5a significantly reduced Min6 cells apoptosis induced by a cocktail of cytokines without a significant effect on the basal rates of apoptosis (Figure 5(d)). Pretreatment of Min6 cells with 0.05 *μ*g/ml exogenous Wnt5a for 48 h decreased proliferation, as assessed by EdU incorporation (Figure 5(e)). Together, these data identified a potential mechanism by which ISCs regulate *β*-cell function via Wnt5a.

### 3.4. Intracellular Mechanism of Action of Wnt5a in the Regulation of Insulin Secretion from Min6 Cells

**Figure 6:**
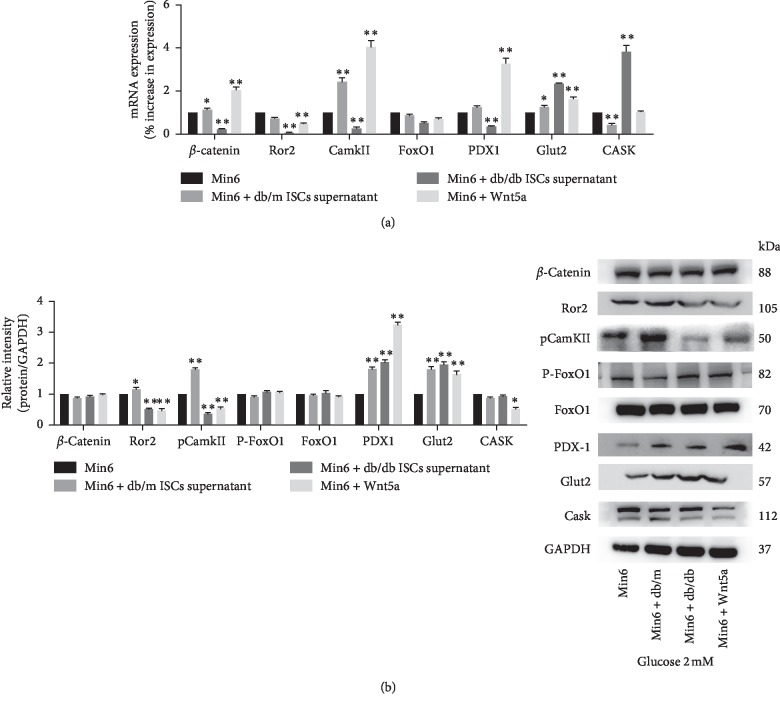
Intracellular mechanism of action of Wnt5a in the regulation of insulin secretion from Min6 cells. (a) GSIS was induced, and qRT-PCR analyses of Min6 cells alone, Min6 cells cultured with ISC supernatant, and Min6 cells cultured with exogenous Wnt5a were performed to measure *β*-catenin, Ror2, CamKII, FoxO1, PDX1, Glut2, and Cask mRNA level (2 mM glucose conditions). ^*∗*^*p* < 0.05 and ^*∗∗*^*p* < 0.01 vs. Min6 cells cultured alone; (b) GSIS was induced, and *β*-catenin, Ror2, pCamKII, pFoxO1, FoxO1, PDX1, Glut2, and Cask protein levels were quantified by western blotting of Min6 cells cultured alone, Min6 cells cultured with ISC supernatant, and Min6 cells cultured with exogenous Wnt5a (2 mM glucose conditions). ^*∗*^*p* < 0.05 and ^*∗∗*^*p* < 0.01 vs. Min6 cells cultured alone.

**Figure 7:**
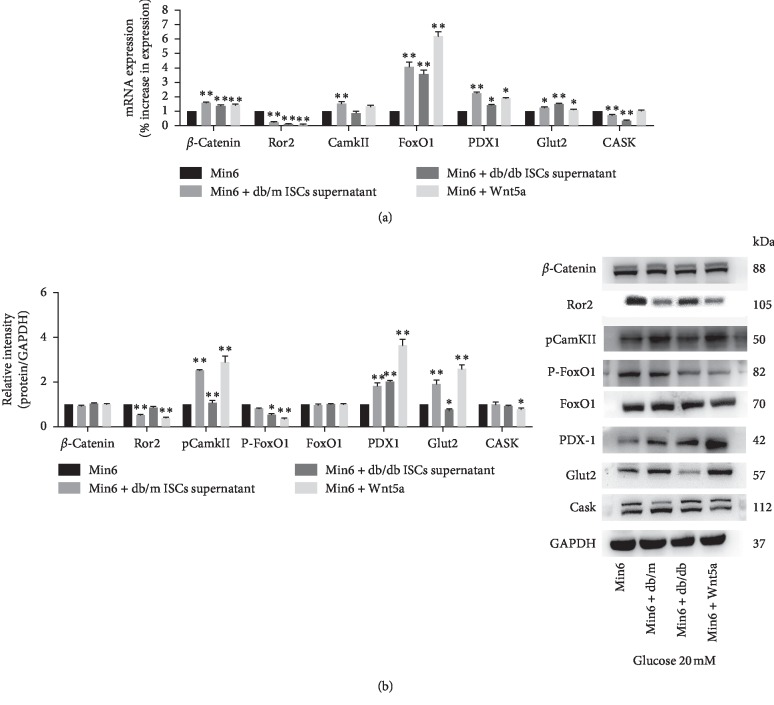
Intracellular mechanism of action of Wnt5a in the regulation of insulin secretion from Min6 cells. (a) GSIS was induced, and qRT-PCR analyses of Min6 cells cultured alone, Min6 cells cultured with ISC supernatant, and Min6 cells cultured with exogenous Wnt5a were performed to measure *β*-catenin, Ror2, CamKII, FoxO1, PDX1, Glut2, and Cask mRNA level (2 mM glucose conditions). ^*∗*^*p* < 0.05 and ^*∗∗*^*p* < 0.01 vs. Min6 cells cultured alone; (b) GSIS was induced, and *β*-catenin, Ror2, pCamKII, pFoxO1, FoxO1, PDX1, Glut2, and Cask protein levels were quantified by western blotting of Min6 cells cultured alone, Min6 cells cultured with ISCs supernatant, and Min6 cells cultured with exogenous Wnt5a (20 mM glucose conditions). ^*∗*^*p* < 0.05, ^*∗∗*^*p* < 0.01 vs. Min6 cells cultured alone.

**Figure 8:**
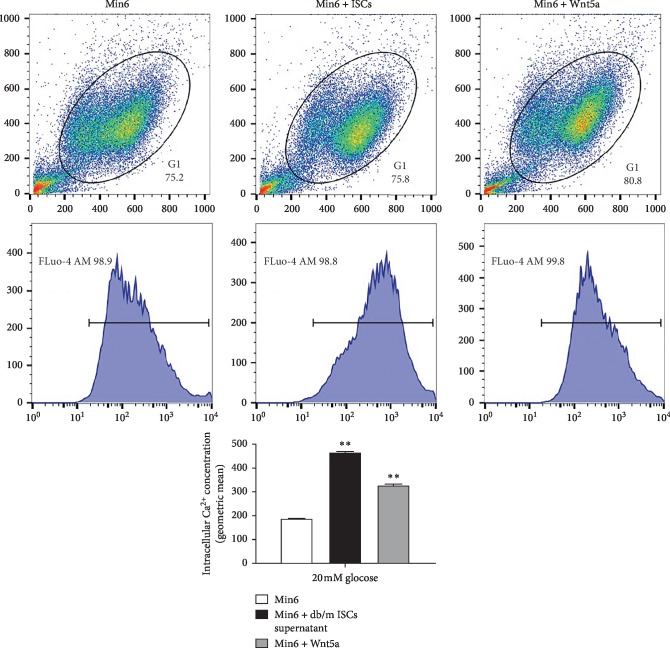
Intracellular Ca^2+^ concentration in Min6 cells. Ca^2+^ change in response to GSIS. Min6 cells cultured with ISC supernatant and with exogenous Wnt5a and loaded with 5 *μ*M Fluo-4 AM. Ca^2+^ was measured in buffer and 20 mM glucose was added to each well. *n* = 3.

To determine whether a change in the Wnt signalling cascades is involved in the Wnt5a-mediated regulation of GSIS in Min6 cells, we compared the expression levels of *β*-catenin, Ror2, and CamKII in Min6 cells. Under 2 mM glucose conditions, we observed an increase in the expression of *β*-catenin and CamKII mRNA but a decrease in the expression of Ror2 in Min6 cells cultured with db/m mouse ISC supernatant or exogenous Wnt5a compared to control Min6 cells (Figure 6(a)). In contrast, the mRNA levels of *β*-catenin, CamKII, and Ror2 were significantly decreased in Min6 cells cultured with db/db mouse ISC supernatant (Figure 6(a)). The changes in the expression of each protein differed slightly from the changes in the expression of the corresponding mRNA. Min6 cells cultured with db/m mouse ISC supernatant had higher protein levels of pCamKII than control Min6 cells and Min6 cells cultured with exogenous Wnt5a, but the *β*-catenin level was not significantly affected (Figure 6(b)). Under stimulation with 20 mM glucose, the CamKII mRNA and pCamKII protein levels were significantly higher, but the Ror2 expression levels were much lower in Min6 cells cultured with db/m mouse ISC supernatant or exogenous Wnt5a than in control Min6 cells (Figure 7). The intracellular Ca^2+^ concentration in Min6 cells increased in cultured in vitro with supernatant from db/m mouse ISCs and exogenous Wnt5a compared to that from control Min6 cells (Figure 8). These results revealed that ISCs regulate Min6 cell insulin secretion through the Wnt5a protein-induced Wnt-calcium pathway.

GSIS in *β*-cells is associated with the FoxO1-PDX1-Glut2-insulin signalling pathway. We hypothesized that Wnt5a regulates GSIS by interacting with this signalling pathway. To test this hypothesis, we compared the expression levels of FoxO1, PDX1, Glut2, and Cask in Min6 cells exposed to different treatments. Under 2 mM glucose conditions, we observed a significant increase in the expression of PDX1 and Glut2 protein and mRNA in Min6 cells cultured with db/m ISC supernatant or exogenous Wnt5a. In contrast, neither the level nor phosphorylation status of the FoxO1 protein changed. The Cask protein level but not the mRNA level decreased in Min6 cells cultured with exogenous Wnt5a. Under stimulation with 20 mM glucose, both the mRNA and protein expression of PDX1 and Glut2 increased significantly in Min6 cells cultured with db/m mouse ISC supernatant or exogenous Wnt5a. Treatment of Min6 cells with db/m mouse ISC supernatant and exogenous Wnt5a significantly increased the mRNA levels of FoxO1 but decreased pFoxO1 phosphorylation. The Cask protein level but not the mRNA level decreased in Min6 cells cultured with exogenous Wnt5a (Figures 6 and 7). Together, the results of these GSIS experiments revealed a potential mechanism by which ISCs regulate *β*-cell function associated with the change in the FoxO1-PDX1-Glut2-insulin signalling cascades.

## 4. Discussion

This study aimed at identifying the effects of ISCs on insulin secretion from Min6 cells and determining the underlying intracellular signalling mechanism. Our data showed that ISCs increased insulin secretion from Min6 cells via Wnt5a. All of these effects were associated with the activation of the Wnt-calcium and FoxO1-PDX1-Glut2-insulin signalling pathways. To our knowledge, this report is the first to show that ISCs regulate the intracellular mechanism of insulin secretion in Min6 cells via the Wnt5a protein.

The cells in the islets of Langerhans have distinct regulatory functions and operate within a complex regulatory network involving paracrine and neuronal control of energy homeostasis [21]. Specifically, *α*-cells, *δ*-cells, and PP cells in the internal microenvironment of islets are involved in the regulation of insulin synthesis and secretion in *β*-cells through paracrine hormones such as glucagon, somatostatin, and amylin [22,23]. Our previous findings suggested that ISCs accelerate activation in the diabetic state, which is characterized by increased *α*-SMA protein expression, accelerated migration, and increased synthesis and secretion of extracellular components and that the Reg1 protein inhibits this activation. We found that insulin secretion from islets cocultured in vitro with db/m mouse ISCs was significantly increased compared to that from islets cocultured with ISCs from db/db mice. Subsequent iTRAQ protein screening of the ISCs showed that both ISCs cultured under static conditions and their supernatants showed high Wnt5a protein expression compared with diabetic ISCs [24–30]. The mechanisms leading to ISCs quiescence play an important role in regulating islet function via the Wnt5a-Fzd5 system and can help us understand the crosstalk between cells inside the islets of Langerhans and serve as a target for the prevention of diabetes.

As a signalling pathway highly conserved throughout evolution, the Wnt signalling pathway plays a key role in pancreatic development in mice, rats, and humans [31–35]. Studies have shown that the Wnt signalling pathway regulates the proliferation of islet *β*-cells and that Wnt3a stimulates insulin secretion in cocultured *β*-cell lines by upregulating the Pitx2 transcription factors [36]. Wnt5a plays important roles in the processes governing embryonic development and pathological disorders, such as cancer [37] and inflammatory disease [38], throughout the lifespan of organisms. Wnt5a-Fzd5 signalling plays a role in proper insulin-induced islet formation and cell migration during pancreatic development in vertebrates [8]. Our recent clinical study showed that Wnt5a levels were significantly downregulated in patients with newly diagnosed T2DM and gradually increased in long-term diabetes patients with kidney disease. Vitamin D3 supplementation improved the serum levels of Sfrp5 and Wnt5a in patients with T2DM [39]. In addition, previous studies indicated that Wnt5a plays an antiproliferative role in INS-1 cells and that Wnt5a promotes glucose-induced insulin secretion from islets [16,40]. Our previous research showed that insulin secretion from islets after coculture with ISCs from Wnt5a^−/−^db/m mice was significantly reduced compared to that after coculture with ISCs transfected with control nontargeting shRNAs [7]. To date, research on the connections among the Wnt5a protein, Sfrp5, and diabetes has mainly focused on adipose tissue, inflammation, and insulin resistance. However, the associations among ISCs, the Wnt5a protein, and *β*-cell function, especially the mechanism by which the Wnt5a protein promotes *β*-cell insulin secretion, have been poorly studied. In our study, insulin secretion levels were increased in Min6 cells after culture with db/m mouse ISC supernatant or exogenous Wnt5a protein, but proliferation and apoptosis were decreased. These experimental results were consistent with those of previous studies. We hypothesize that the Wnt5a protein participates not only in the development of the pancreas but also in the regulation of insulin secretion in *β*-cells. Wnt5a may thus be an important mediator of crosstalk between ISCs and *β*-cells.

The findings of this study also provided insight into the intracellular mechanisms associated with Wnt5a-mediated increases in insulin secretion from Min6 cells. In our study, Min6 cells cultured with db/m mouse ISC supernatant or exogenous Wnt5a had higher levels of pCamKII than control Min6 cells, and decreased expression of Ror2 did not affect the expression of the *β*-catenin, demonstrating that the increase in insulin secretion-induced culture with db/m mouse ISC supernatant was caused by the effect of the Wnt5a protein on the noncanonical Wnt-calcium signalling pathway. Previous studies have confirmed that Wnt5a not only affects noncanonical signalling pathways such as the Wnt/calcium signalling pathway but also affects Wnt/*β*-catenin signalling as a typical protein in the Wnt noncanonical signalling pathway [41]. Our results confirmed that Wnt5a promotes the activation of the Wnt-calcium signalling pathway to further increase the calcium ion concentration in Min6 cells and reduce the expression of Ror2. These experimental results are consistent with those of previous studies. Many genes are related to the insulin secretion from *β*-cells, for example, *β*-cell transcription factors (PDX1, Rfx6, and NeuroD1); insulin gene transcription factors; genes mediating GSIS, such as FoxO1, PDX1, Glut2, Gck, Abcc8, and Kcnj11; and genes mediating insulin granule exocytosis (Cask) [42–45].

The FoxO1-PDX1-Glut2-insulin signalling pathway is an important mechanism regulating insulin secretion [19]. FoxO1 has been confirmed as an important factor for the physiological adaptation of *β*-cell mass and function [46]. Transgenic overexpression of FoxO1 in the *β*-cells results in defective GSIS and impaired glucose tolerance in mice [47]. The binding of FoxO1 to the PDX1 promoter negatively regulates the transcription of this gene, suggesting that the expression of PDX1 is regulated by of FoxO1 [48]. The expression of PDX1 is significantly reduced in diabetic mouse models. PDX1 not only is involved in islet development but also can bind to the A3 transcriptional regulatory region of the insulin gene and activate its transcription, thus upregulating insulin expression [49]. Studies have indicated that PDX1 is a master transcription factor in the regulation of Glut2 and that genetic deletion of *β*-cell-specific PDX1 in mice drastically and selectively reduces the expression of Glut2 [50]. Our study showed that the Wnt5a protein can enhance the expression of PDX1 and Glut2 and decrease the expression of pFoxO1. These results revealed that Wnt5a decreases FoxO1 phosphorylation and subsequently increases the expression of PDX1 and Glut2 to maintain both the glucose-sensing ability of Min6 cells and systemic glucose tolerance. These experimental results were consistent with those of previous studies. Moreover, our results revealed inconsistencies in FoxO1 gene and protein expression, which we believe result from the diversity of posttranscriptional translation. In our future studies, we will aim at identifying the mechanisms underlying this difference. Previous studies have shown that FoxO1 inhibits insulin granule exocytosis through downregulation of Cask expression in INS-1 cells [45]. Our study confirmed that the Wnt5a protein can suppress the expression of pFoxO1, but the expression of Cask did not increase accordingly. We hypothesized that the duration of Min6 cell exposure to high glucose conditions might be a critical factor affecting Cask expression and may have generated experimental results differing from those of previous studies. Although these results clearly supported our hypothesis that ISCs are important for the maintenance of insulin secretion in a physiological environment, the specific mechanism underlying the effect of Wnt5a on the insulin secretion pathway still needs further exploration; Biochip technology and transgenic models could be used to elucidate further insights into the pathogenesis of T2DM.

## 5. Conclusion

In conclusion, we investigated a type of ISC and found that this population of ISCs regulates insulin secretion from *β*-cells via the Wnt5a protein; we demonstrated the specific mechanisms by which novel ISC-derived secreted products affect *β*-cell function by activating the Wnt-calcium signalling pathway and increasing the expression of insulin regulatory proteins. These results can help us to clarify the pathogenesis of diabetes and to identify new targets in order to improve diabetes treatment.

## Figures and Tables

**Table 1:** The PCR primer sequences.

Gene	Primer	Sequence	PCR product length
*β*-catenin	*β*-catenin-F	TTTCCCAGTCCTTCACGC	112
	*β*-catenin-R	ATGCCCTCATCTAGCGTCTC	

Ror2	Ror2-F	GGTTGTGCAAGAGCCACGA	117
	Ror2-R	CCGTTGGTAGCCACACACTG	

CamKII	CamKII-F	TTTGTCCGTGGAATGTGG	98
	CamKII-R	CGTGACGCGAGTACATAGGT	

FoxO1	FoxO1-F	AGGATGACCTGGGAGATGG	225
	FoxO1-R	GCGGTGCAAACGAATAGC	

PDX-1	PDX-1-F	GAGGTGCTTACACAGCGGAA	117
	PDX-1-R	GGGGCCGGGAGATGTATTT	

Glut2	Glut2-F	TCAGAAGACAAGATCACCGGA	78
	Glut2-R	GTCATAGCCGAACTGGAAGGA	

Cask	Cask-F	CCTACCCCAACCTCCCCAT	109
	Cask-R	TGCAGTGTAGTAACAGCACAG	

## Data Availability

The data used to support the findings of this study are available from the corresponding author upon request.
